# Supplementary material for: Cities: Complexity, theory and history
Source: PLoS One. 2020 Dec 8;15(12):e0243621. doi: 10.1371/journal.pone.0243621 (PMC7723246; doi:10.1371/journal.pone.0243621)
Supplement: Attachment — Submitted filename: Response to reviews.docx [file pone.0243621.s001.docx]

11/2/2020

Dear Editors:

Thank you for soliciting three helpful reviews of our article. Before proceeding to address the reviewers' comments we want to re-emphasize that our intention was to submit a manuscript for consideration of the special issue on cities and complexity. We communicated our intention to do so to Marta Gonzalez and Diego Rybski, the two guest editors of the planned special issue. In writing the submitted paper we intended the audience to be general urban scientists and not necessarily archaeologists. So although we are pleased that the archaeologists who reviewed our paper found that the data and analysis supported our conclusions, we would appreciate it if the submission was considered for the special issue and if it were to be shared with the two guest editors.

Reviewer #1:

The major question for the paper is whether it needs the long general discussion that is front-loaded, in which 'what is a city?' and why theory might be useful are discussed. For my own part, I thought that these could be dispensed with and the paper could start more or less at p. 9.

- We thank the reviewer for pointing out that the initial sections of our paper are longer than they should be. However, we do not think cutting these passages entirely is appropriate because we wrote our paper for the special collection “cities and complexity” and hope it will be read by urbanists interested in complexity, and complexity scientists interested in cities. Still, we take the reviewer’s comment as an indication that we could do more to connect these general considerations to the analysis and results. So we have restructured and shortened the initial sections of the paper in an effort to accomplish this.

Reviewer #2:

The analysis presented by Ortman, Lobo, and Smith of archeological, historic, and contemporary settlement data demonstrates very effectively the consistent impact of distance-interaction principles on the internal residential density of settlements across a wide range of population sizes, time periods, and technological regimes. The authors do not describe what they do in these terms, and some readers might find an easier path into the abstract mathematical treatment of the subject if they did.

- We are very pleased that the reviewer is convinced by our analyses of the archaeological and OECD data, and we thank this person for pointing out that distance-interaction principles are an additional way in which one could introduce our approach. Since the paper is intended primarily for the community of researchers interested in cities and complexity, we think the existing approach is more appropriate for this paper. But we will certainly keep this idea in mind for future efforts geared toward different audiences.

For Ortman, Lobo, and Smith, it is the somewhat cryptically named "pre-factor" that puts a number on the circumstances that vary across space and time and can cause settlements with larger populations to be less densely packed or even more densely packed than expected on the basis of the average tendency.

- We thank the reviewer for pointing out that our terminology may appear cryptic to some readers, and we have added a few comments in the relevant places in an effort to clarify the meaning of the term “pre-factor”.

This number does point clearly to such "deviant" settlement systems and identify the direction and strength of their differences from the "normal" relationship between population size and residential density, but because the general relationship is established in terms of such broad averaging, much potentially important variation is subsumed within the averages (and thus lost sight of), and the "pre-factor," in itself, does little to help us actually understand variation.

- We agree that the pre-factor of a scaling relation is a measure of a system-level property that does not contribute to the understanding of variation across individual settlements within the system. However, the pre-factor does help one identify which sorts of variation are significant, given the properties of the system. We have added language to the discussion to emphasize this further.

And there is, indeed, a methodological issue here, leading to concepts like Metropolitan Statistical Areas or Functional Urban Areas for contemporary cities, and arbitrary rules setting 100 m as the size of gaps in surface artifact distributions that indicate separate archaeological sites which are then automatically assumed to be separate social communities.

Drennan, Berrey, and Peterson (2015) are cited as justification for this archeological rule, although actually it is argued there (pp. 52-61) that this is an arbitrary and misleading approach that can split socially meaningful settlements into parts or even create "settlements" where none exist.

The fact that it is very hard to draw a line around settlements in some systems is not just a methodological challenge to get around; it is a message about substance. The cases enumerated in the previous paragraph are ones in which the social phenomenon of "settlement" and the interaction that constitutes one are truly different in character—more diffuse and less bounded in reality than in cases where the boundaries of settlements are pretty clear. In extreme cases there may be no local community structure at all, just scattered farmsteads. Insisting on seeing all settlement systems in terms of clearly bounded settlement units that correspond to bounded local interaction communities blinds us to important aspects of interaction structure. And, well, this is but one of the directions that exploring deviations from Ortman, Lobo, and Smith's general averages can productively go.

- This is an excellent point and we have inserted some discussion to this effect in the paper.

The complete supporting data are not actually available in the tDAR repository cited.

- We were waiting to post the data until our paper was closer to acceptance, but we have done so now.

Reviewer #3:

As a minor recommendation, I think the paper would benefit from less direct quotes; some of the existing ones could easily be paraphrased and/or shortened.

- We have removed some of the direct quotes as part of re-structuring the initial part of the paper, but we feel the ones that remain are important because they provide strong justification for our approach.

The second part of the bibliography is confusing, why are the references in the section "References added by Jose" not incorporated into the general bibliography?

- We thank the reviewer for pointing out this formatting issue. We have fixed it in the revised version.
